# Interleukin‐11 receptor is an alternative α‐receptor for interleukin‐6 and the chimeric cytokine IC7

**DOI:** 10.1111/febs.17309

**Published:** 2024-10-29

**Authors:** Hendrik T. Weitz, Julia Ettich, Puyan Rafii, Christoph Wittich, Laura Schultz, Nils C. Frank, Denise Heise, Matthias Krusche, Juliane Lokau, Christoph Garbers, Kristina Behnke, Doreen M. Floss, Harald Kolmar, Jens M. Moll, Jürgen Scheller

**Affiliations:** ^1^ Institute of Biochemistry and Molecular Biology II, Medical Faculty and University Hospital Düsseldorf Heinrich Heine University Düsseldorf Germany; ^2^ Institute of Clinical Biochemistry Hannover Medical School Germany; ^3^ Institute for Organic Chemistry and Biochemistry Technical University of Darmstadt Germany; ^4^ Centre of Synthetic Biology Technical University of Darmstadt Germany

**Keywords:** cross‐talk, cytokine, gp130, IL‐11, interleukin 6

## Abstract

The cytokine interleukin 6 (IL‐6) signals via the IL‐6 α‐receptor (IL‐6Rα or IL‐6R) in complex with the gp130 β‐receptor. Cell type restricted expression of the IL‐6R limits the action of IL‐6 mainly to hepatocytes and some immune cells. Here, we show that IL‐6 also binds to the IL‐11 α receptor (IL‐11Rα or IL‐11R) and induces signaling via IL‐11R:gp130 complexes, albeit with a lower affinity compared to IL‐11. Antagonistic antibodies directed against IL‐11R, but not IL‐6R, inhibit IL‐6 signaling via IL‐11R:gp130 receptor complexes. Notably, IL‐11 did not cross‐react with IL‐6R. IL‐11R has also been identified as an alternative α receptor for the CNTF/IL‐6‐derived chimeric cytokine IC7, which has recently been shown to induce weight loss in mice. Accordingly, the effects of therapeutic monoclonal antibodies against IL‐6 or IL‐6R, which both block IL‐6 signaling, may be slightly different. These findings provide new insights into IL‐6 signaling and therefore offer new potential therapeutic intervention options in the future.

## Graphical abstract

The major pro‐inflammatory cytokine interleukin‐6 (IL‐6) contributes to regeneration and tissue remodeling. IL‐6 signals via the IL‐6 α‐receptor in complex with the gp130 β‐receptor. Here, we show that IL‐6 also binds to the IL‐11 α‐receptor and induces signaling via gp130 homodimers, albeit with a lower affinity compared to IL‐11. These findings provide new insights into IL‐6 signaling and therefore offer new potential therapeutic intervention options in the future, particularly in the application of antibodies that block IL‐6 signaling either by binding to IL‐6 or to the IL‐6 α‐receptor.

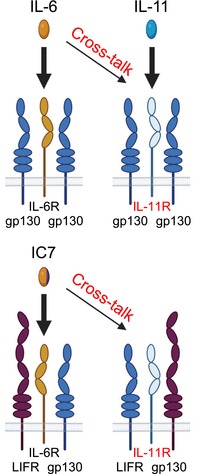

**Abbreviations**
HIL‐6/HIL‐11 or cHIL‐6: (chimeric) hyper‐cytokine
IL: interleukin
IL‐11R: IL‐11 α receptor
IL‐6R: IL‐6 α‐receptor
RFP: receptor fusion protein
sgp130: soluble gp130

## Introduction

The IL‐6 family of cytokines consists of nine members, cardiotrophin‐1 (CT‐1), cardiotrophin‐like cytokine (CLC), ciliary neurotrophic factor (CNTF), IL‐6, IL‐11, IL‐27, IL‐31, leukemia inhibitory factor (LIF), and oncostatin M (OSM) [1, 2, 3], which all activate central signaling cascades including the JAK/STAT, ERK and AKT pathways [4, 5]. Apart from IL‐31, all other IL‐6‐type cytokines induce signal transduction via homodimers of gp130 plus α‐receptor chains for IL‐6, IL‐11 (IL‐6Rα or IL‐11Rα, respectively), or heterodimers of gp130:LIF receptor (LIFR) for CLC, CT‐1, CNTF, LIF and OSM, gp130:OSM‐receptor (OSMR), and gp130:IL‐27 receptor (IL‐27R) for IL‐27 [4, 6]. The IL‐6‐type cytokines IL‐6, IL‐11, and CNTF first form heterodimeric complexes with specific non‐signal transducing α‐receptors to acquire the binding affinity for their signal transducing β‐receptors, namely IL‐6Rα, IL‐11Rα, and CNTFRα, respectively. While the β‐receptor gp130 is almost ubiquitously expressed, the limited expression of the α‐receptor confers the cellular specificity of IL‐6, IL‐11, and CNTF. Notably, CNTF binds the IL‐6R as a CNTFR alternative α‐receptor, albeit with lower affinity [2]. In addition, human OSM can signal via two different β‐receptor combinations, gp130:OSMR and gp130:LIFR [2]. The IL‐27 cytokine subunit p28 was also shown to bind not only its canonical non‐signaling alpha receptor EBI3, but also the IL‐6 receptor [7]. In general, receptor cross‐talk broadens the number of cells that can be activated by individual cytokines. We and others have found phenotypic differences between IL‐6 and IL‐6R‐deficient mice [8, 9]. IL‐6‐deficient mice showed slightly attenuated dextran sodium sulphate (DSS)‐induced colitis, whereas the phenotype of IL‐6R‐deficient mice was indistinguishable from that of wild‐type mice [9]. In addition, epidermal wound healing was reduced in IL‐6 knock‐out mice but not in IL‐6R knock‐out mice [8]. Recently, IL‐6R‐deficient mice were found not to gain more body weight on a high‐fat diet, as previously described for IL‐6‐deficient mice [10, 11]. These differences may indicate the presence of alternative ligands for IL‐6R or receptors for IL‐6. Whether these or other differences also exist in humans is currently unknown, but is likely to be elucidated as larger cohorts of patients are treated not only with IL‐6R but also with IL‐6 neutralizing antibodies. IL‐6 is involved in a variety of physiological processes such as hematopoiesis, the innate and adaptive immune system, metabolism, neural development and survival, but may also play a critical role in disorders such as inflammatory diseases (e.g. rheumatoid arthritis, inflammatory bowel disease) or the development and maintenance of cancer [12]. While IL‐11 was originally described for its lymphopoietic and hematopoietic properties, it also induces the acute phase response in hepatocytes, controls bone homeostasis, skull formation, and fibrosis [13].

Both IL‐6 and IL‐11 contribute to regeneration and tissue remodeling [14]. Deficiency of either IL‐6R or IL‐11R is associated with severely impaired regeneration processes [8, 9, 15] or impaired bone formation and fertility [16, 17].

Taken together, some data suggest receptor cross‐talk of IL‐6. Structurally and functionally, IL 11R is the closest homolog to IL 6R within the IL‐6 family. In addition, IL‐6 and IL‐11 share some structural homology and are the only members of the IL‐6 family of cytokines that signal via gp130 homodimers [1]. To date, IL‐11R‐activated signaling via IL‐6 has not been described, which may be explained by the insufficient concentrations required to induce such cross‐talk [18]. Here we show that IL‐6 can bind to the membrane‐bound and soluble IL‐11R, albeit with lower efficiency compared to IL‐6R. Consequently, IL‐6 induces signaling via IL‐11R:gp130 receptor complexes. These results demonstrate an IL‐6R‐independent activation of the gp130/STAT3 axis by IL‐6 through the IL‐11R.

## Results

### hIL‐6 uses hIL‐11R for non‐canonical gp130 signaling

**Fig. 1:**
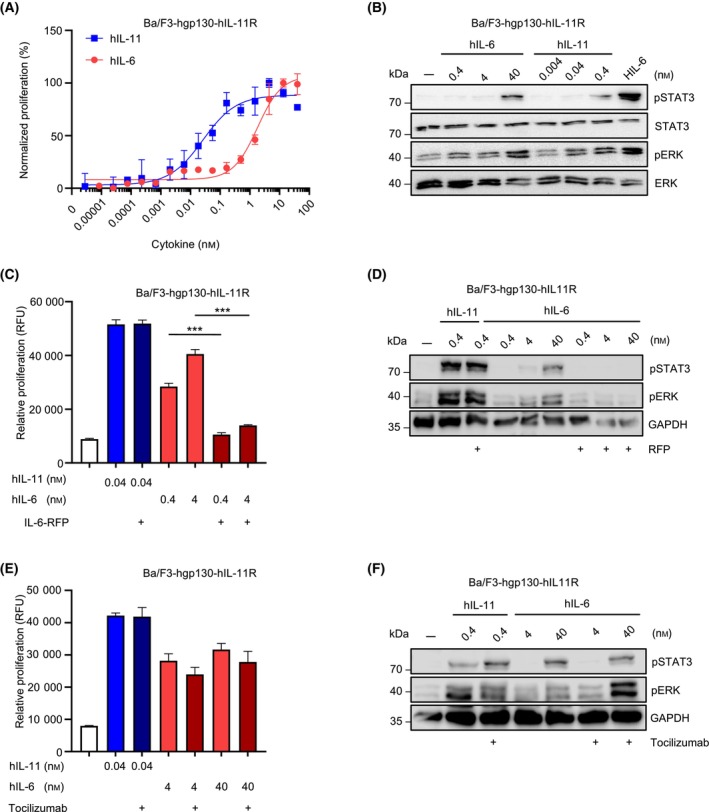
Non‐canonical gp130 receptor complex formation of human IL‐6 via the IL‐11 receptor. (A) Ba/F3 cells stably transduced with hgp130 and human IL‐11R (Ba/F3‐hgp130‐hIL‐11R) were incubated with the indicated concentrations of hIL‐6ts or hIL‐11ts. Cellular proliferation assay was performed in triplicate and determined after 72 h as described in experimental procedures. Error bars indicate the standard deviation (SD). One representative experiment out of three independent experiments is shown. (B) Ba/F3‐hgp130‐hIL‐11R cells were incubated with the indicated concentrations of hIL‐6ts, hIL‐11his and Hyper IL‐6 (HIL‐6, 10 ng·mL^−1^) for 20 min. STAT3 and ERK (phosphorylation) were determined by Western blotting as described in experimental procedures. One representative experiment out of three independent experiments is shown. (C) Ba/F3 cells stably transduced with hgp130 and human IL‐11R (Ba/F3‐hgp130‐hIL‐11R) were incubated with the indicated concentrations of hIL‐6ts and hIL‐11ts and the IL‐6‐RFP‐inhibitor (in 5 : 1 molar ratio). Cellular proliferation assay was performed in triplicate and determined after 72 h as described in experimental procedures. Error bars indicate the standard deviation (SD). One representative experiment out of three independent experiments is shown. Statistical analysis used unpaired *t* test, ***P ≤ 0.001. (D) Ba/F3‐hgp130‐hIL‐11R cells were incubated with the indicated concentrations of hIL‐6ts, hIL‐11ts, and the RFP‐inhibitor for 20 min. STAT3 and ERK phosphorylation was determined by western blotting as described in experimental procedures. One representative experiment out of three independent experiments is shown. (E) Ba/F3‐hgp130‐hIL‐11R cells were incubated with the indicated concentrations of hIL‐6ts, hIL‐11ts and 0.6 μm IL‐6R mAb (tocilizumab). Cellular proliferation assay was performed in triplicate and determined after 72 h as described in experimental procedures. Error bars indicate the standard deviation (SD). One representative experiment out of three independent experiments is shown. (F) Ba/F3‐hgp130‐hIL‐11R cells were incubated with the indicated concentrations of hIL‐6ts, hIL‐11ts and Tocilizumab for 20 min. STAT3 and ERK phosphorylation was determined by western blotting as described in experimental procedures. One representative experiment out of three independent experiments is shown.

To analyze whether IL‐6 transduces signals via IL‐11R and gp130, we used Ba/F3 cells stably transduced with human gp130 (hgp130) and human IL‐11R (hIL‐11R), which canonically signal and proliferate in the presence of human IL‐11 (hIL‐11). [19]. We found that, in addition to hIL‐11, human IL‐6 (hIL‐6) induced proliferation of Ba/F3 hgp130 hIL‐11R cells in a dose‐dependent manner (Fig. 1A). The half‐maximal effective concentration (EC50) for hIL‐6 needed to induce cellular proliferation of Ba/F3‐hgp130‐hIL‐11R cells was 2.31 ± 0.55 nm and about 100fold higher compared to hIL‐11 (EC50 = 21.58 ± 4.31 pm) (Fig. 1A, Table 1). Consequently, around 100fold higher concentrations of hIL‐6 compared to hIL‐11 were required to induce STAT3 and ERK phosphorylation in Ba/F3‐hgp130‐hIL‐11R cells (Fig. 1B). Signaling was dependent on hIL‐11R, because hIL‐6 induced proliferation and STAT3/ERK phosphorylation of Ba/F3‐hgp130‐hIL‐6R cells (EC50 = 13.29 ± 14.25 pm, Table 1) but failed to activate Ba/F3‐hgp130 cells lacking hIL‐11R expression (Fig. S1A–D).

**Table 1:** EC50s in arithmetic means ± SD to induce proliferation of Ba/F3 cell lines.

Ba/F3 cell lines	EC50 in arithmetic means ± SD		
Ba/F3‐hgp130‐hIL‐11R	hIL‐6ts: 2.31 ± 0.55 nm	hIL‐11ts: 21.58 ± 4.31 pm	
Ba/F3‐hgp130‐mIL‐11R	hIL‐6ts: 2.16 ± 1.07 nm	hIL‐11ts: 0.22 ± 0.13 nm	
Ba/F3‐hgp130	HIL‐6ts: 46.61 ± 8.13 pm	HIL‐11ts: 57.76 ± 7.14 pm	cHIL‐6ts: 194.03 ± 53.52 pm
Ba/F3‐hgp130‐hIL‐6R‐hLIFR	IC7Fc: 38.42 ± 19.78 pm		
Ba/F3‐hgp130‐hIL‐11R‐hLIFR	IC7Fc: 7.87 ± 1.70 nm		
Ba/F3‐hgp130‐hIL‐6R	hIL‐6ts: 13.29 ± 14.25 pm		
Ba/F3‐hgp130	hIL‐6ts: none		
Ba/F3‐hgp130‐hIL‐6R	hIL‐11ts: none		
Ba/F3‐hgp130‐mIL‐11R	mIL‐11ts: 132.20 ± 85.74 pm	mIL‐6ts: none	

The non‐canonical receptor complex formation of hIL‐6 was verified using reagents blocking signaling via hIL‐11R:hgp130, and hIL‐6R:hgp130 receptor complexes. First of all, hIL‐6‐induced proliferation was analyzed in the presence of the IL‐6‐specific inhibitor IL‐6R fusion protein (IL‐6‐RFP) [20]. IL‐6‐RFP is a fusion protein of the cytokine‐binding domains of gp130 fused to the soluble IL‐6R (sIL‐6R) which specifically binds and neutralizes IL‐6 activity [20]. Therefore, IL‐6‐RFP reduced hIL‐6‐ but not hIL‐11‐induced proliferation of Ba/F3‐hgp130‐hIL‐11R cells (Fig. 1C). Moreover, STAT3 and ERK phosphorylation in Ba/F3‐hgp130‐hIL‐11R cells induced by hIL‐6 but not by IL‐11 was blocked by a molar excess of IL‐6‐RFP (Fig. 1D). As expected, the neutralizing IL‐6R antibody tocilizumab did not inhibit hIL‐6‐ and hIL‐11‐induced proliferation and STAT3 and ERK phosphorylation of Ba/F3‐hgp130‐hIL‐11R cells (Fig. 1E,F) but IL‐6‐induced proliferation of Ba/F3‐hgp130‐hIL‐6R cells (Fig. S1E).

Next, the affinity (*K*_D_) of 22.26 nm for hIL‐6 binding to the soluble hIL‐11R was determined by surface plasmon resonance (Table 2, Fig. S2A), which was about 10fold and 40fold lower compared to binding of hIL‐6 to hIL‐6R (*K*_D_ = 2.29 nm) and hIL‐11 to hIL‐11R (*K*_D_ = 0.53 nm), respectively (Table 2, Fig. S2B,C). We also observed binding of hIL‐11 to hIL‐6R with a *K*_D_ of 58.37 nm (Table 1, Fig. S2D). However, hIL‐11 largely failed to induce cellular proliferation of Ba/F3‐hgp130‐hIL‐6R cells in a concentration range up to 360 nm (EC50 n.d.) or STAT3 phosphorylation (Fig. S3A,B), suggesting that hIL‐11 is not able to signal via hIL‐6R:hgp130 complexes.

**Table 2:** Binding kinetics data from SPR of the cytokines IL‐6, IL‐11 and IC7 to human sIL‐6R and human sIL‐11R.

	sIL‐6R			sIL‐11R		
	IL‐6	IL‐11	IC7	IL‐6	IL‐11	IC7
*K*_D_ (nm)	2.29	58.37	6.38	22.26	0.53	48.14
*k*_a1_ (Ms^−1^)	2.324E+05	1.641E+05	9.215E+04	6.604E+04	2.302E+06	5.990E+04
*k*_a2_ (Ms^−1^)	3.076E‐03	1.118E‐02	7.505E‐03	6.572E‐03	4.538E‐03	1.040E‐02
*k*_d1_ (s^−1^)	5.555E‐03	9.246E‐02	9.316E‐03	1.294E‐02	6.915E‐03	1.932E‐02
*k*_d2_ (s^−1^)	3.266E‐04	1.292E‐03	5.056E‐04	8.569E‐04	9.721E‐04	1.825E‐03

### Non‐canonical gp130 receptor complex formation of human IL‐6 via murine IL‐11 receptor was inhibited by neutralizing IL‐11R monoclonal antibody

**Fig. 2:**
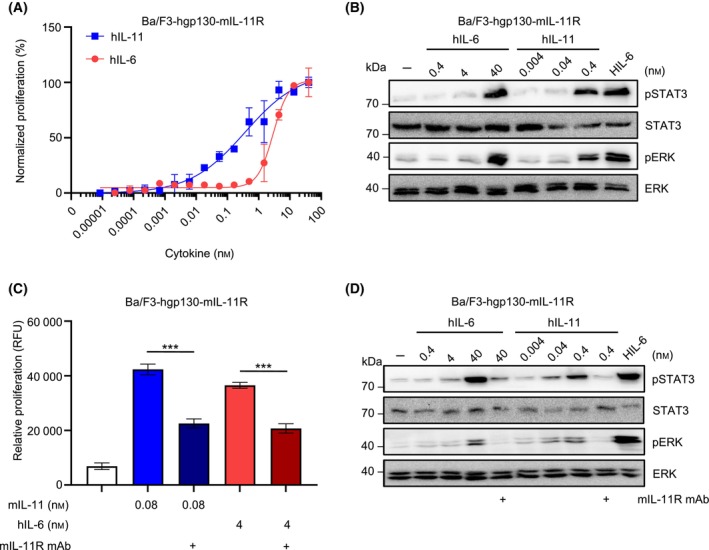
IL‐6 signaling via IL‐11R in blocked by IL‐11R directed antibodies. (A) Ba/F3 cells stably transduced with hgp130 and murine IL‐11R (Ba/F3‐hgp130‐mIL‐11R) were incubated with the indicated concentrations of hIL‐6ts or hIL‐11ts. Cellular proliferation assay was performed in triplicate and determined after 72 h as described in experimental procedures. Error bars indicate the standard deviation (SD). One representative experiment out of three independent experiments is shown. (B) Ba/F3‐hgp130‐mIL‐11R cells were incubated with the indicated concentrations of hIL‐6ts, hIL‐11his and HIL‐6 (10 ng·mL^−1^) for 20 min. STAT3 and ERK (phosphorylation) were determined by western blotting as described in experimental procedures. One representative experiment out of three independent experiments is shown. (C) Ba/F3 cells stably transduced with hgp130 and murine IL‐11R (Ba/F3‐hgp130‐mIL‐11R) were incubated with the indicated concentrations of hIL‐6ts, mIL‐11ts and mIL‐11R mAb (0.2 μm). Cellular proliferation assay was performed in triplicate and determined after 72 h as described in experimental procedures. Error bars indicate the standard deviation (SD). One representative experiment out of three independent experiments is shown. Statistical analysis used unpaired *t* test, ***P ≤ 0.001. (D) Ba/F3‐hgp130‐mIL‐11R cells were incubated with the indicated concentrations of hIL‐6ts, hIL‐11ts and HIL‐6 (10 ng·mL^−1^) and mIL‐11R mAb (0.2 μm) for 20 min. STAT3 and ERK (phosphorylation) was determined by western blotting as described in experimental procedures. One representative experiment out of three independent experiments is shown.

hIL‐6 also induced proliferation of Ba/F3‐hgp130 cells expressing the murine IL‐11R (mIL‐11R) in a dose‐dependent manner with an EC50 of 2.16 ± 1.07 nm which was 10fold higher compared to Ba/F3‐hgp130‐mIL‐11R cells stimulated with hIL‐11 (EC50 = 0.22 ± 0.13 nm) (Table 1, Fig. 2A). Accordingly, about 100fold higher concentrations of hIL‐6 compared to hIL‐11 were required to induce STAT3 and ERK phosphorylation of Ba/F3‐hgp130‐mIL‐11R cells (Fig. 2B).

To block IL‐11R specifically, we stimulated Ba/F3‐hgp130‐mIL‐11R cells with mIL‐11 in the presence and absence of a neutralizing mIL‐11R monoclonal antibody (mAb). We used Ba/F3‐hgp130 cells expressing mIL‐11R and not the hIL‐11R, because neutralizing antibodies for the hIL‐11R were not commercially available. The antagonistic mIL‐11R mAb, however, blocked the proliferation of Ba/F3‐hgp130‐mIL‐11R cells induced by hIL‐6 and mIL‐11 (Fig. 2C). STAT3 and ERK phosphorylation in Ba/F3‐hgp130‐mIL‐11R cells induced by hIL‐11 or by hIL‐6 was also inhibited by the mIL‐11R mAb (Fig. 2D). Here, we used hIL‐6 because in contrast to mIL‐11, mIL‐6 failed to induce proliferation and STAT3 phosphorylation of Ba/F3‐hgp130‐mIL‐11R cells (EC50 mIL‐11: 132.20 ± 85.74 pm, EC50 mIL‐6: none) (Table 1, Fig. S3C,D). Taken together, our data demonstrated cross‐talk of IL‐6 via IL‐11R:gp130 complexes.

### An IL‐6:soluble IL‐11R fusion protein induces trans‐signaling

**Fig. 3:**
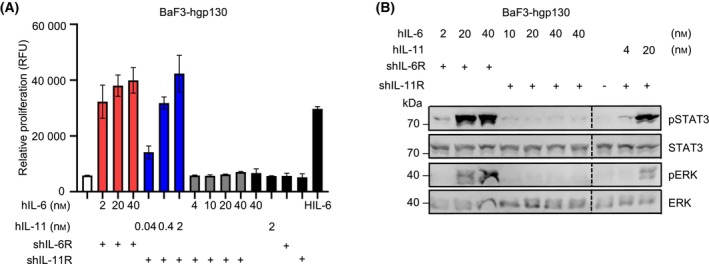
IL‐6 and sIL‐11R fail to induce trans‐signaling. (A) Ba/F3‐hgp130 cells were incubated with the indicated concentrations of IL‐6ts or IL‐11ts and fixed concentrations of shIL‐6R and shIL‐11R (4.4 nm). Cellular proliferation assay was performed in triplicate and determined after 48 h as described in experimental procedures. Error bars indicate the standard deviation (SD). One representative experiment out of three independent experiments is shown. (B) Ba/F3‐hgp130 cells were incubated with the indicated concentrations of IL‐6ts or IL‐11ts and fixed concentrations of sIL‐6R and sIL‐11R (4.4 nm). STAT3 and ERK (phosphorylation) was determined by western blotting as described in experimental procedures. One representative experiment out of three independent experiments is shown.

To analyze whether IL‐6 can induce trans‐signaling via IL 6:sIL‐11R complexes, we co‐stimulated cells with mixtures of hIL‐6 and soluble hIL‐11R (shIL‐11R). Here, concentrations of up to 40 nm hIL‐6 with 4.4 nm shIL‐11R failed to induce cell proliferation and STAT3 phosphorylation (Fig. 3A,B), whereas the same concentrations for IL‐6:sIL‐6R and IL‐11:sIL‐11R results in cellular proliferation (Fig. 3A). We hypothesize that the affinity of hIL‐6 towards shIL‐11R might be too low to induce trans‐signaling for the conditions used here.

**Fig. 4:**
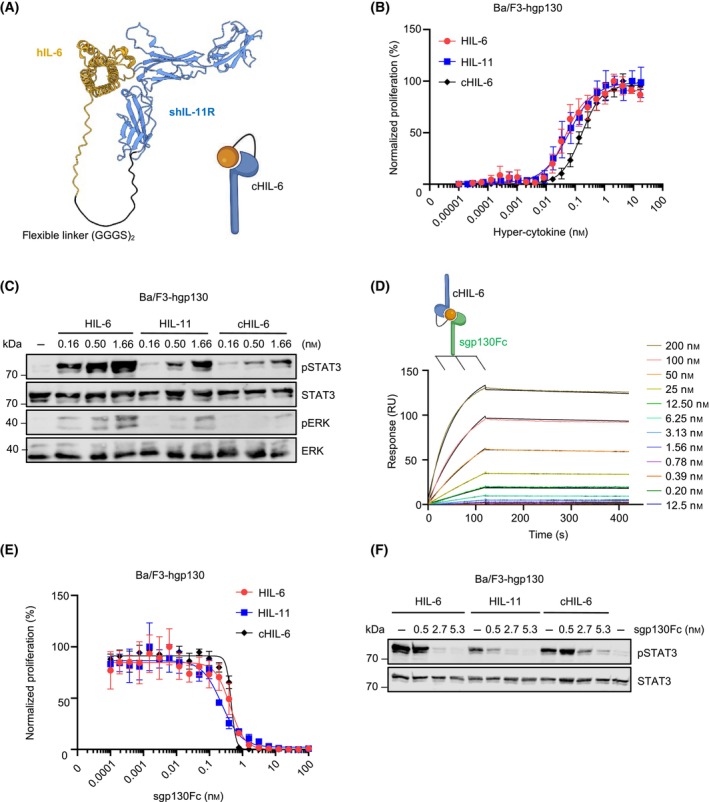
The fusion protein of IL‐6 and sIL‐11R executes trans‐signaling. (A) 3D‐Model of chimeric hyper‐IL‐6 plus a corresponding schematic illustration of cHIL‐6, consisting of sIL‐11R fused together with IL‐6 via a flexible peptide linker. The structure of cHIL‐6 was predicted by AlphaFold2 (ColabFoldv1.5.5) and visualized by chimera. (B) Ba/F3‐hgp130 cells were incubated with the indicated concentrations of HIL‐6, HIL‐11, and cHIL‐6. Cellular proliferation assay was performed in triplicate and determined after 72 h as described in experimental procedures. Error bars indicate the standard deviation (SD). One representative experiment out of three independent experiments is shown. (C) Ba/F3‐hgp130 cells were incubated with the indicated concentrations of HIL‐6, HIL‐11, and cHIL‐6 for 20 min. STAT3 and ERK (phosphorylation) was determined by western blotting as described in experimental procedures. One representative experiment out of three independent experiments is shown. (D) SPR analysis of cHIL‐6 binding to sgp130Fc. Sgp130Fc was immobilized on a Protein‐A chip and increasing concentrations of the cytokine were injected. Sensorgrams in response units (RU) over time are depicted as colored lines, and global fit data are displayed as black lines. (E) Ba/F3‐hgp130 cells were incubated with the indicated concentrations of HIL‐6, HIL‐11, and cHIL‐6 and sgp130Fc. Cellular proliferation assay was performed in triplicate and determined after 48 h as described in experimental procedures. Error bars indicate the standard deviation (SD). One representative experiment out of three independent experiments is shown. (F) Ba/F3‐hgp130 cells were incubated with a constant concentration 1.6 nm of HIL‐6, HIL‐11, and cHIL‐6 and the indicated concentrations of sgp130Fc for 20 min. STAT3 and ERK (phosphorylation) was determined by western blotting as described in experimental procedures. One representative experiment out of three independent experiments is shown.

The fusion proteins of hIL‐6 and soluble hIL‐6R (Hyper‐IL‐6, HIL‐6) and hIL‐11 and shIL‐11R (Hyper‐IL‐11, HIL‐11) were previously described as synthetic cytokines that mimic trans‐signaling by activation of cells expressing only gp130. Hyper‐cytokines have been reported to be 10–100× more potent trans‐signaling inducers compared to the naturally occurring transient cytokine:soluble‐cytokine‐receptor complexes [21, 22, 23]. To analyze whether in principle IL‐6:sIL‐11R complexes induce trans‐signaling, we engineered the synthetic chimeric Hyper‐cytokine IL‐6/sIL‐11R (cHIL‐6), which is a fusion protein composed of the N‐terminally located soluble hIL‐11R connected to hIL‐6 via a flexible (GGGS)_2_ peptide linker followed by a C‐terminal Twin‐Strep‐tag® purification tag (Fig. 4A). cHIL‐6 was expressed in Expi293‐F cells and purified from the cell culture supernatant by affinity chromatography. cHIL‐6 as well as other used recombinant proteins were validated by gel electrophoresis (Fig. S4A–D).

The biological activity of cHIL‐6 was determined and compared to HIL‐6, and HIL‐11 using Ba/F3‐hgp130 cells. HIL‐6, HIL‐11, and cHIL‐6 induced cellular proliferation with EC50 of 46.61 ± 8.13 pm, 57.76 ± 7.14 pm, 194.03 ± 53.52 pm, respectively (Fig. 4B, Table 1). Consequently, concentrations of around 1 nm of HIL‐6, HIL‐11, and cHIL‐6 induced sustained STAT3 and ERK phosphorylation (Fig. 4C). Binding kinetics of cHIL‐6, HIL‐6 and HIL‐11 to soluble gp130 (sgp130) were determined by surface plasmon resonance using immobilized sgp130Fc, which is a fusion protein of soluble hgp130 to the Fc‐part of an IgG antibody [24]. *K*_D_ of 243.7 pm for HIL‐6 to sgp130Fc, 396.5 pm for HIL‐11 to sgp130Fc and of 1.24 nm for cHIL‐6 to sgp130Fc (Fig. 4D, Table 3, Fig. S4E,F) were in good agreement with the activity differences observed in cellular assays. While dissociation rates of the three hyper‐cytokines were similarly low, differences in the overall *K*_D_ mostly relied on the more efficient association rate constants ranking from HIL‐6 over HIL‐11 to cHIL‐6 (Table 3). sgp130 is the natural inhibitor of IL‐6 and IL‐11 trans‐signaling [24]. To test, if sgp130Fc also inhibits IL‐6:sIL‐11R complexes, Ba/F3‐gp130 cells were stimulated with constant amounts of HIL‐6, HIL‐11 and cHIL‐6 (1.7 nm) and increasing concentrations of sgp130Fc. sgp130Fc efficiently inhibited HIL‐6‐, HIL‐11‐ and cHIL‐6‐induced cellular growth (IC50 of sgp130Fc for HIL‐6: 428 ± 88 pm; for HIL‐11: 213 ± 53 pm; for cHIL‐6: 324 ± 205 pm) (Fig. 4E) and STAT3 phosphorylation (Fig. 4F), defining sgp130Fc as efficient inhibitor of IL‐6/sIL‐11R trans‐signaling.

**Table 3:** Binding kinetics data from SPR of the hyper‐cytokines HIL‐6, HIL‐11 and cHIL‐6 to sgp130Fc.

	sgp130Fc		
	HIL‐6	HIL‐11	cHIL‐6
*K*_D_ (nm)	0.2437	0.3965	1.239
*k*_a_ (Ms^−1^)	1.476E+06	3.595E+05	9.226E+04
*k*_d_ (s^−1^)	3.465E‐04	1.425E‐04	1.144E‐04

In conclusion, our data showed that a fusion protein of IL‐6 and sIL‐11R efficiently induce trans‐signaling.

### Non‐canonical gp130 receptor complex formation of the chimeric cytokine IC7 via the IL‐11 receptor

**Fig. 5:**
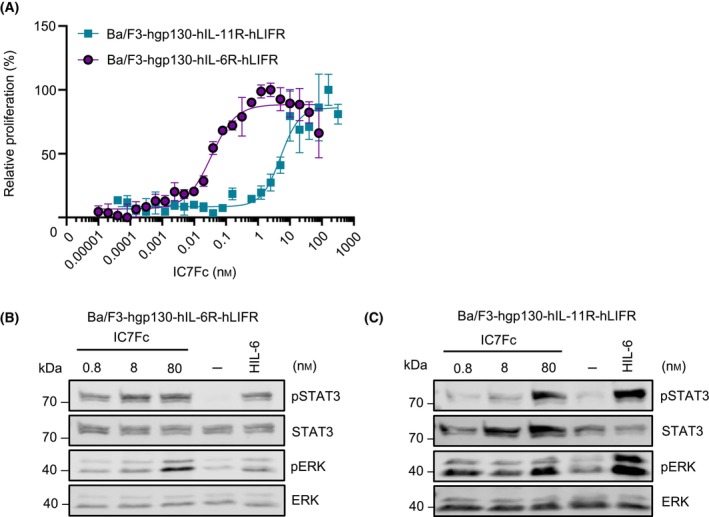
Non‐canonical gp130 receptor complex formation of the cytokimera IC7 via the IL‐11 receptor. (A) Ba/F3‐hgp130‐hIL‐6R‐hLIFR and Ba/F3‐hgp130‐hIL‐11R‐hLIFR cells were incubated with the indicated concentrations of IC7Fc. Cellular proliferation assay was performed in triplicate and determined after 72 h as described in experimental procedures. Error bars indicate the standard deviation (SD). One representative experiment out of four independent experiments is shown. (B, C) Ba/F3‐hgp130‐hIL‐6R‐hLIFR and Ba/F3‐hgp130‐hIL‐11R‐hLIFR cells were incubated with the indicated concentrations of IC7Fc, HIL‐6 (10 ng·mL^−1^) and unstimulated (−) for 20 min. STAT3 (phosphorylation) was determined by western blotting as described in experimental procedures. One representative experiment out of three independent experiments is shown.

Chimeric cytokines use the framework of natural cytokines with at least one receptor binding site transferred from a closely related cytokine [25, 26, 27]. The chimeric cytokine IC7 was generated by replacing the gp130 binding site III of IL‐6 [25] with the LIFR binding site III of CNTF [28], resulting in signaling via the non‐natural receptor complex of IL‐6R, LIFR and gp130 [25]. The dimeric fusion protein IC7Fc was recently shown to improve glucose tolerance and hyperglycemia, thereby preventing weight gain and liver steatosis in mice [29]. Here, we tested whether IC7Fc also transduced signals via gp130:IL‐11R:LIFR complexes. Therefore, we compared the cytokine‐induced proliferation of Ba/F3‐hgp130‐hIL‐6R‐hLIFR, and Ba/F3‐hgp130‐hIL‐11R‐hLIFR cells. Proliferation of Ba/F3‐hgp130‐hIL‐11R‐hLIFR, and Ba/F3‐hgp130‐hIL‐6R‐hLIFR cells was induced by IC7Fc in a dose‐dependent manner (Fig. 5A). EC50 for IC7Fc for the stimulation of cellular proliferation for Ba/F3‐hgp130‐hIL‐6R‐hLIFR cells was 38.42 ± 19.78 pm compared to 7.87 ± 1.70 nm for Ba/F3‐hgp130‐hIL‐11R‐hLIFR cells (Table 1). As expected from IL‐6, also around 100fold higher concentrations of IC7Fc were required to induce STAT3‐phosphorylation in Ba/F3‐IL‐11R‐gp130‐LIFR cells compared to Ba/F3‐IL‐6R‐gp130‐LIFR cells (Fig. 5B,C). The *K*_D_ for the binding of IC7 to the sIL‐6R and sIL‐11R were 6.38 and 48.14 nm, respectively, as determined by surface plasmon resonance (Table 2, Fig. S5A,B). In summary, our data showed IL‐11R cross‐talk for the chimeric cytokine IC7Fc.

## Discussion

This study has three major findings. First of all, we identified the IL‐11R as alternative α‐receptor for IL‐6, albeit with a lower association constant compared to the canonical α‐receptor IL‐6R. Second, the IL‐6‐sIL‐11R fusion protein cHIL‐6 facilitates trans‐signaling. Third, the chimeric cytokine IC7 also uses the IL‐11R as a low‐affinity α‐receptor, thereby signaling via IL‐11R:gp130:LIFR complexes.

Compared to IL‐11, approximately 100‐fold higher concentrations of IL‐6 were required to achieve comparable signaling strength via IL‐11R:gp130 complexes, due to the lower affinity of IL‐6 for IL‐11R compared to IL‐11. The overall binding affinity of IL‐11 to IL‐11R (*K*_D_ = 0.53 nm) was ~ 40fold higher in comparison than IL‐6 to IL‐11R (*K*_D_ = 22.6 nm). Previously, affinities in the range of 10 and 50 nm were reported for the interaction of IL‐11 and sIL‐11R [30, 31] which is lower compared to our results.

Our data largely excluded the possibility of natural trans‐signaling via IL‐6:sIL‐11R complexes, suggesting that the affinity of IL‐6 for IL‐11R is mainly sufficient for classic signaling via membrane‐bound IL‐11R:gp130 receptor complexes. However, using the novel designer cytokine cHIL‐6, we have shown that, at least in principle, the cross‐talk also extends to trans‐signaling via the sIL‐11R, as has been shown for IL‐11 [19] and corresponding Hyper IL‐11 variants [22, 30, 32].

Among the α‐receptor dependent cytokines of the IL‐6 type cytokine family, which include IL‐6, IL‐11, CNTF, Cytokine‐Like Factor‐1 (CLF‐1) and p28, up to now cross‐talk was described for CNTF and p28. As shown by Schuster *et al*. [33], CNTF uses either the high‐affinity CNTF receptor (CNTFR) or the low‐affinity IL‐6R as α‐receptor to induce signaling via complex formation with gp130 and LIFR. The concentrations of 4–40 nm IL‐6 which were required to induce signaling via IL‐11R:gp130 complexes are comparable to those required for CNTF cross‐talk via IL‐6R. Although serum IL‐6 levels are in the low pg·mL^−1^ range in healthy individuals, IL‐6 is strongly upregulated to the ng·mL^−1^ range in inflammatory conditions [34] and even higher in septic conditions [35, 36, 37, 38]. In addition, paracrine signaling, where the cytokine‐producing cell is in close contact with the recipient cell, should allow even higher local concentrations of IL‐6 to be achieved, suggesting that IL‐6 signaling via IL‐11R may also be present under physiological conditions *in vivo*.

Recently, there has been some interest in the chimeric IL‐6‐like cytokine IC7 for its ability to improve glucose tolerance and hyperglycemia, which in turn prevents weight gain and hepatic steatosis in mice [29]. Based on the structure of IL‐6, IC7 has replaced the gp130 binding site III of IL‐6 with the LIFR receptor binding site III of CNTF [6], resulting in the activation of the non‐natural IL‐6R:LIFR:gp130 receptor complex. Here, we show that IC7 has cross‐talk activity towards the IL‐11R:LIFR:gp130 receptor complex, which may be important for a full understanding of the mode of action of IC7 in food intake and weight regulation.

Both IL‐6 and IL‐11 are required for regenerative processes and tissue remodeling [15, 39]. Interestingly, unlike IL‐6 and IL‐11R‐deficient mice, IL‐6R‐deficient mice did not have wound‐healing defects [8, 9, 15]. In the inflammatory environment present during wound healing, high levels of IL‐6 are secreted. Local concentrations of IL‐6 within the wounded tissue may allow IL‐6‐mediated IL‐11R activation at the wound site. One role of IL‐6 cross‐talk may be the induction of IL‐11R‐mediated STAT3 phosphorylation as an early response to tissue injury to accelerate tissue repair and remodeling through maximal gp130 activation. This may also be of interest for the application of antibodies that block IL‐6 signaling either by binding to IL‐6 or to the IL‐6R. The effects of the approved therapeutic monoclonal antibodies Siltuximab directed against IL‐6 or Tocilizumab and Sarilumab directed against IL‐6R might differ slightly.

## Materials and methods

### Cells and reagents

Ba/F3‐hgp130 and variants thereof were generated via retroviral transduction as described previously [26, 40, 41]. All Ba/F3 cell lines were mycoplasma‐free and grown in Dulbecco's Modified Eagle Medium (DMEM) high glucose culture medium (Gibco, Life Technologies, Grand Island, NY, USA) supplemented with 10% fetal bovine serum (FCS), penicillin (60 mg·L^−1^) and streptomycin (100 mg·L^−1^). Ba/F3‐hgp130 cells were grown in the presence of 10 ng·mL^−1^ recombinant Hyper IL‐6 (HIL‐6), which is a fusion protein of the human sIL‐6R and human IL‐6 connected with a flexible peptide linker. HIL‐6 was expressed and purified as described [21, 42]. Ba/F3‐hgp130‐hIL‐6R cells were grown in the presence of 10 ng·mL^−1^ recombinant hIL‐6 and Ba/F3‐hgp130‐hIL‐11R cells were cultured in the presence of 10 ng·mL^−1^ recombinant hIL‐11, respectively. Ba/F3‐hgp130 cells were grown in the presence of 10 ng·mL^−1^ recombinant HIL‐6. All cells used in this study were incubated at 37 °C with 5% CO_2_ in a water‐saturated atmosphere. The IL‐6R monoclonal antibody (mAb) tocilizumab (ACTEMRA/RoACTEMRA®) was obtained from Roche (Basel, Switzerland). Anti‐murine IL‐11R antibody was bought (#AF490; R&D Systems, Inc., Minneapolis, MN, USA). For western blotting antibodies directed against STAT3 phosphorylated at Tyr705 (clone D3A7), STAT3 (clone 124H6), ERK (clone L34F12) and ERK phosphorylated at Thr202/Tyr204 (clone D13.14.4E) were obtained from Cell Signaling Technology (Frankfurt, Germany). For Fc‐tag detection after protein purification rabbit human IgG‐Fc HRP conjugate mAb (#31423) was obtained from Invitrogen (Thermo Fisher Scientific, Invitrogen AG, Carlsbad, CA, USA). For Twin‐Strep‐tag® detection after protein purification StrepMAB‐Classic‐HRP (#2‐1509‐001) was obtained from Iba Lifesciences GmbH (Göttingen, Germany).

### Detection of mRNAs for murine gp130, murine IL‐6R and murine IL‐11R in Ba/F3 cell lines

RNA purification from Ba/F3 cell lines was performed according to the manufacturer's instructions (NucleoSpin® RNA; MACHEREY‐NAGEL GmbH & Co. KG, Düren, North Rhine‐Westphalia, Germany). Gene expression of target genes was performed by iTaq Universal SYBR Green One‐Step Kit (Bio‐Rad, Großkugel, Saxony‐Anhalt, Germany). For analysis, the expression levels of all target genes were normalized to the expression of β‐actin (Δcycle threshold). Gene expression values were calculated based on the Δ*C*_t_ method. Relative quantities (RQs) were determined using the equation: $\mathrm{RQ}=2^{-\Delta C_{t}}$. Primer sequences were (5′–3′): β‐actin‐fw TGACAGGATGCAGAAGGAGA, β‐actin‐rev CGCTCAGGAGGAGCAATG, mgp130‐fw TACCTCAAACAAGCCGCTC, mgp130‐rev CCCATCTTTCCCACCTTCATC, mIL‐6R‐fw CCTGAGACTCAAGCAGAAATGG, mIL‐6R‐rev AGAAGGAAGGTCGGCTTCAGT, mIL‐11R‐fw CCCGCTACCTTACTTCCTAC, mIL‐11R‐rev TACTCACTCCAGAACTCTGCC. Ba/F3 cells lack mRNAs coding for murine gp130, murine IL‐6R and murine IL‐11R. Only cells, ectopically expressing murine gp130, murine IL‐6R and murine IL‐11R proteins as have mRNAs coding for murine gp130, murine IL‐6R and murine IL‐11R (Fig. S6A–C).

### Construction of expression plasmids for IL‐6/IL‐11 and variants thereof

pcDNA3.1 expression plasmids for HIL‐6, hIL‐6R and hIL‐11R were described previously [21, 40]. For the cDNA coding of the IL‐6/sIL‐11R fusion protein (cHIL‐6), the sequence coding for soluble IL‐11R of amino acids 23–317 was amplified by polymerase chain reaction (PCR) and cloned into pcDNA3.1‐HIL‐6Fc resulting in pcDNA3.1‐cHIL‐6Fc with an exchange of IL‐6R by IL‐11R. The cDNA encoding hIL‐11 was synthesized by GeneArt AG (Regensburg, Germany) and subcloned into the pcDNA3.1‐cHIL‐6Fc resulting in pcDNA3.1‐HIL‐11Fc with an exchange of IL‐6 by IL‐11. Next, all cDNAs coding for hyper‐cytokines were subcloned into a Twin‐Strep‐tag® (ts) containing pcDNA3.1 plasmid via restriction enzymes resulting in variants HIL‐6ts, cHIL‐6ts, and HIL11ts. The cDNA coding for murine IL‐6ts was synthesized by Biocat (Heidelberg, Germany) and subcloned into a Twin‐Strep‐tag® containing pcDNA3.1 plasmid.

### Expression and purification of proteins

His‐tagged human IL‐11 was produced in and purified from *Escherichia coli*. sgp130Fc was produced in stably transfected CHO‐K1 cells using a roller bottle system and hyper‐cytokines were produced in Expi293F™ cells (Thermo Fisher Scientific, Invitrogen AG) following the manufacturer's protocol. All other proteins (Twin‐strep‐tagged human and murine IL‐6 (hIL‐6ts, mIL‐6ts), Twin‐strep‐tagged human and murine IL‐11 (hIL‐11ts, mIL‐11ts), Fc‐tagged IL‐6 (IL‐6Fc), Fc‐tagged IC7 (IC‐7Fc)) were produced in Expi293F™ or Expi CHO‐S cells (Thermo Fisher Scientific, Invitrogen AG) following the manufacturer's protocol. Culture supernatants from roller bottles and Expi‐culture flasks were harvested and centrifuged at 1000 ***g*** and 4 °C for 30 min, followed by centrifugation of the resulting supernatant at 10 000 ***g*** at 4 °C for 30 min. The supernatant of the second centrifugation step was filtered (bottle top filter, 0.45‐μm pore diameter; Nalgene, Rochester, NY, USA) and purified by affinity chromatography, as already described in Berg *et al*. [43].

IL‐6‐RFP was kindly gifted from Prof. Dr. G. Müller‐Newen (Institute of Biochemistry RWTH, Aachen, Germany) [44]. Purification of Fc‐tagged proteins: Supernatants containing Fc‐tagged protein were loaded on a protein‐A column (HiTrap MabSelect PrismA 1 mL; GE Healthcare Düsseldorf, North Rhine‐Westphalia, Germany) at a flow rate of 1 mL·min^−1^. After loading, the column was washed with 30× column volumes of phosphate buffered saline (PBS) (137 mm NaCl, 2.7 mm KCl, 12 mm HPO_4_^2−^/H_2_PO^4−^, pH 7.4). Fc‐proteins were eluted at pH 3.2–3.5 using a 50 mm citric acid buffer. Elution fractions were collected and fractions containing the protein peak were pooled, and pH was adjusted to 7 with 1 m Tris buffer. Purification of Twin‐Strep‐tag®‐proteins: Twin‐Strep‐tag® proteins were purified with a Strep‐Tactin®XT 4Flow® FPLC column 1 mL (Iba Lifesciences) and loaded at a flow rate of 1 mL·min^−1^. After loading, the column was washed with 30× column volumes of PBS. Twin‐Strep‐tag® proteins were eluted with a biotin buffer. Elution fractions were collected. Fractions containing the protein peak were pooled. After affinity chromatography, proteins were buffer exchanged to PBS using illustra NAP25 (GE Healthcare Life Sciences, Munich, Germany) columns. Protein concentrations were determined by measuring absorbance at 280 nm, and samples were flash‐frozen in liquid nitrogen. Protein quality was assessed by sodium dodecyl sulfate–polyacrylamide gel electrophoresis (SDS/PAGE) following Coomassie staining and western blotting, as already described in Heise *et al*. [45].

### Proliferation assays

Proliferation of Ba/F3 cell lines was performed as described previously [46]. Ba/F3 cells were washed three times with PBS, and 50 000 cells·mL^−1^ were cultured for 3 days in a final volume of 100 μL/well in the presence of cytokines and inhibitors in a 96‐well plate. The CellTiter‐Blue® Reagent was used to determine cellular viability by recording the fluorescence (excitation 560 nm, emission 590 nm) using an Infinite M200 PRO plate reader (Tecan, Crailsheim, Germany) immediately after adding 20 μL of reagent per well (time point 0) and up to 120 min thereafter. Extinction was determined with the following parameters: excitation wavelength 590 nm, emission wavelength 590 nm. Relative fluorescence units (RFU) were calculated by subtraction of time point zero values. If samples were normalized to either highest or lowest means of triplicates, or to negative (unstimulated) or positive controls (10 ng·mL^−1^ HIL‐6). All experiments were performed in three independent replicates.

### Cytokine stimulation for western blotting

For analysis of signal transduction via STAT3, pSTAT3, ERK and pERK, Ba/F3 cell lines were washed five times with PBS and subsequently starved for 4 h in serum‐free DMEM. Following starvation, 1 × 10^6^ cells were incubated at 37 °C with the respective cytokine variants, receptors, hyper‐cytokines or inhibitors for 20 min and harvested by centrifugation (400 ***g***, 4 °C, 5 min) following snap freezing of the cell pellet in liquid nitrogen. Cell pellets were lysed, and protein concentration was determined via bicinchoninic acid (BCA) assay (Thermo Fisher Scientific, Invitrogen AG). Fifty microgram of total lysate protein was separated by SDS/PAGE. For western blotting using fluorescent‐labeled antibodies: Proteins on SDS/PAGE gels were transferred to Nitrocellulose membrane (Amersham Protan, Cytiva Germany GmbH, Dreieich, Hesse, Germany) for 30 min by using the Trans‐Blot Turbo Transfer System from Bio‐Rad (München, Germany). Membranes were blocked for 1 h with blocking buffer (Intercept® Blocking Buffer; LI‐COR Biosciences GmbH, Bad Homburg, Hesse, Germany) and probed with the primary antibodies (STAT3, pSTAT3, ERK, pERK, all obtained from cell signaling, in 1 : 1000 dilution) over night. After washing, membranes were either incubated with secondary fluorescent‐labeled antibodies IRDye® 800CW donkey anti‐rabbit, and IRDye® 680RD donkey anti‐mouse in 1 : 10 000 dilution for 1 h, washed followed by signal detection using LI‐COR Odyssey (LI‐COR Biosciences GmbH; Model 2800). Secondary antibodies were detected simultaneously on different channels. Data analysis was conducted using image studio lite 5.2 (LI‐COR Biosciences GmbH). For western blotting using fluorescent‐labeled antibodies: Proteins on SDS/PAGE gels were transferred to PVDF membrane using a Trans‐Blot Turbo transfer system (Bio‐Rad). The membrane was blocked in 5% fat‐free dried skimmed milk in TBS‐T (10 mm Tris/HCl, pH 7.6, 150 mm NaCl, 0.05% Tween 20) and then probed with the primary antibody (1 : 1000) in 5% fat‐free dried skimmed milk in TBS‐T (STAT3, Erk) or 5% BSA in TBS‐T (pSTAT3, pErk) at 4 °C overnight. The blots were washed and incubated with the secondary peroxidase‐conjugated antibody (1 : 5000) or streptavidin‐HRP for 1 h before applying the Immobilon Western Chemiluminescent HRP Substrate (Merck Chemicals GmbH, Darmstadt, Hesse, Germany). The ChemoCam Imager (INTAS Science Imaging Instruments GmbH, Göttingen, Germany) was used for signal detection according to the manufacturer's instructions, as already described in Floss *et al*. [41].

### Surface plasmon resonance

For surface plasmon resonance (SPR) experiments, the Biacore X100 instrument (Cytiva Life Sciences, Cytiva Germany GmbH) was used. Analysis was performed in multi‐cycle mode using a NTA sensor chip (BR100407; Cytiva Life Sciences, Cytiva Germany GmbH). Experiments were carried out at 25 °C in PBS with 0.05% (v/v) surfactant P20 (GE Healthcare Düsseldorf) containing 50 μm EDTA. Recombinant his‐tagged soluble hIL‐11R (cat no. 8895‐MR; R&D Systems, Inc.) and soluble hIL‐6R (cat. no. 10537; R&D Systems, Inc.) were immobilized to a single flow cell at a level of 100–250 response units per cycle. Three samples containing only running buffer were injected over both ligand and reference flow cell, followed by the tested analytes serially diluted in the indicated concentrations ranging from 133.33 to 0.183 nm, with an independent final replicate of 4.938 nm. The cytokines were injected at a flow rate of 30 μL·min^−1^ for 180 s, and the dissociation was measured for 800 s. Alternatively, for hyper‐cytokine kinetics, sgp130Fc was coupled via its Fc‐tag on a Protein A Sensor chip (cat no. 29127558; Cytiva). After immobilization of sgp130Fc to a single flow cell at a level of 200–300 response units per cycle, the corresponding hyper‐cytokine was injected at a flow rate of 30 μL·min^−1^ for 120 s, and the dissociation was measured for 300 s in different concentrations as indicated (from 200 to 0.20 nm for cHIL‐6, from 25 to 0.05 nm for HIL‐6 and 50 to 0.62 nm for HIL‐11). The resulting data were reference subtracted and fit to a 1 : 1 binding model for hyper‐cytokines and sgp130Fc kinetics and a two‐state reaction fit for hIL‐6ts, hIL‐11ts, IC7ts, on shIL‐6R and shIL‐11R, using the biacore X100 evaluation software v2.0.1 (Cytiva Germany GmbH).

### Molecular modeling

Interactive visualization and alignment of cytokines was performed with ucsf chimera [version 1.15; Resource for Biocomputing, Visualization, and Informatics (RBVI) at the University of California, San Francisco, CA, USA]. For *in‐silico* subcloning, prediction and creation of plasmid maps snapgene® (version 3.2.1) was used. The structure of cHIL‐6 was predicted by AlphaFold2 (ColabFoldv1.5.5) [47] and visualized by chimera.

### Statistics

Graphs and statistics were created with graphpad prism 8.0.2 (GraphPad Software Inc., Dotmatics, Boston, MA, USA). EC50 and IC50 data are provided as arithmetic means ± SD. Statistics were determined with unpaired *t* tests and statistical significance was set at the level of *P* < 0.05 (*P < 0.05; **P < 0.01; and ***P < 0.001).

## Conflict of interest

The authors declare no conflict of interest.

## Author contributions

HTW, JE, PR, KB designed and performed experiments, analyzed data and wrote the manuscript. JE, PR, CW, LS, NCF, DH, MK, JL, CG performed experiments. HK provided cytokines and wrote the manuscript. DMF, PR and JE provided cell lines. HTW, JE, CW and PR analyzed data. JMM and JS initialized and supervised the study, designed experiments, and wrote the manuscript.

### Peer review

The peer review history for this article is available at https://www.webofscience.com/api/gateway/wos/peer‐review/10.1111/febs.17309.

## Supporting information


**Fig. S1.** IL‐6 did induce proliferation of Ba/F3‐hgp130‐hIL‐6R but not on Ba/F3‐hgp130 cells.
**Fig. S2.** Binding kinetics of IL‐6 and IL‐11 to IL‐6R and IL‐11R, respectively.
**Fig. S3.** IL‐11 did not induce proliferation of Ba/F3‐hgp130‐IL‐6R cells.
**Fig. S4.** Validation of purified recombinant Proteins and binding kinetics of Hyper‐cytokines.
**Fig. S5.** Binding kinetics of IC7 on IL‐6R and IL‐11R, respectively.
**Fig. S6.** Detection of mRNAs for murine gp130, murine IL‐6R and murine IL‐11R in Ba/F3 cell lines by quantitative PCR.

## Data Availability

Data available on request from the authors: The data that support the findings of this study are available from the corresponding author jscheller@uni-duesseldorf.de upon reasonable request.
