# Supplementary material for: Interleukin‐11 receptor is an alternative α‐receptor for interleukin‐6 and the chimeric cytokine IC7
Source: FEBS J. 2024 Oct 29;292(3):523–36. doi: 10.1111/febs.17309 (PMC11796321; doi:10.1111/febs.17309)
Supplement: Supplementary file 1 — Fig. S1. IL‐6 did induce proliferation of Ba/F3‐hgp130‐hIL‐6R but not on Ba/F3‐hgp130 cells. Fig. S2. Binding kinetics of IL‐6 and IL‐11 to IL‐6R and IL‐11R, respectively. Fig. S3. IL‐11 did not induce proliferation of Ba/F3‐hgp130‐IL‐6R cells. Fig. S4. Validation of purified recombinant Proteins and binding kinetics of Hyper‐cytokines. Fig. S5. Binding kinetics of IC7 on IL‐6R and IL‐11R, respectively. Fig. S6. Detection of mRNAs for murine gp130, murine IL‐6R and murine IL‐11R in Ba/F3 cell lines by quantitative PCR. [file FEBS-292-523-s001.pdf]

# Supplemental Figure 1

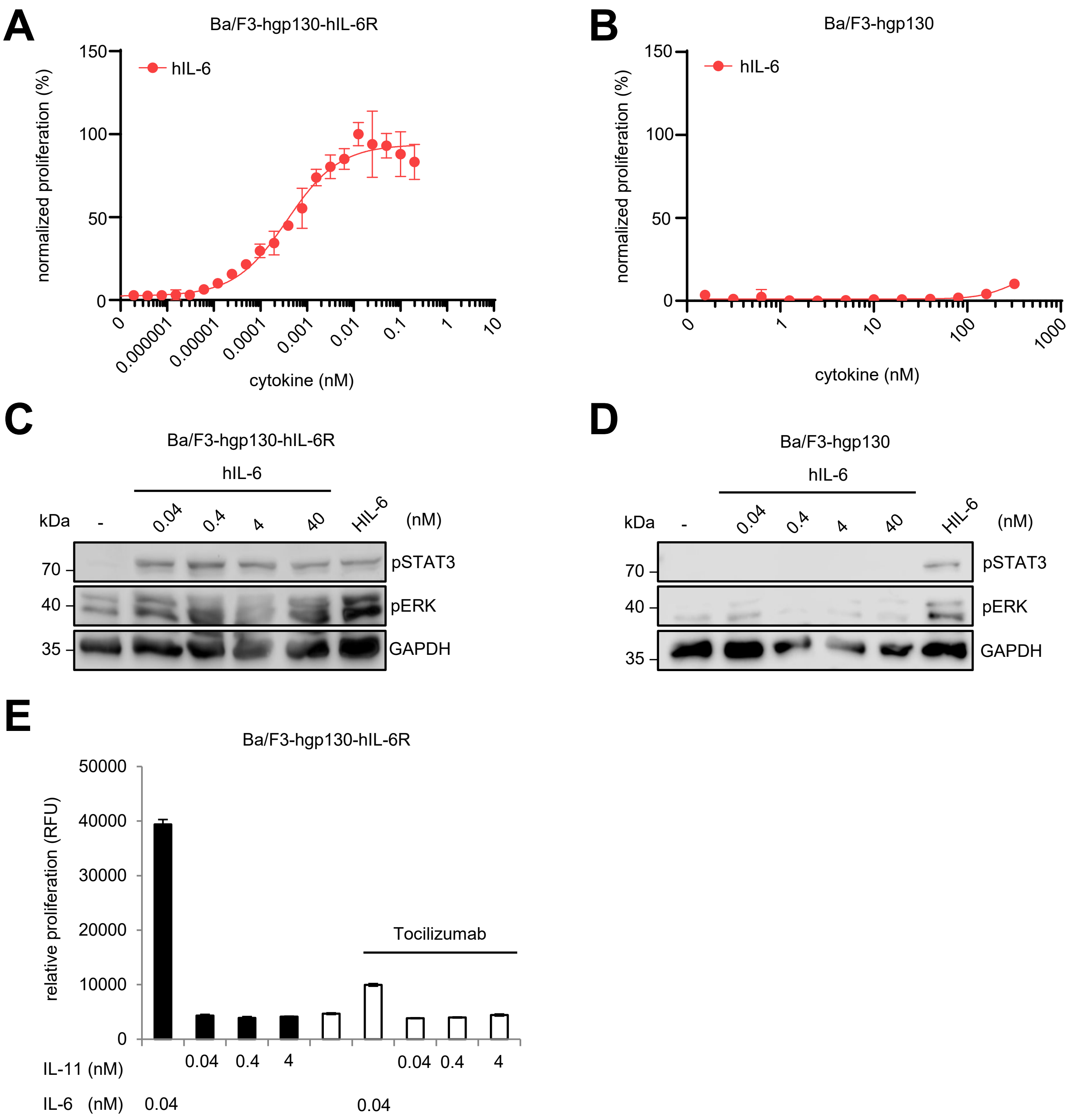


**Supplemental Figure S1: IL-6 did induce proliferation of Ba/F3-hgp130-hIL-6R but not on Ba/F3-hgp130 cells. (A, B)** Ba/F3-hgp130-hIL-6R and Ba/F3-hgp130 cells were incubated with the indicated concentrations of IL-6ts. Cellular proliferation assay was performed in triplicate and determined after 72 h as described in experimental procedures. One representative experiment out of three independent experiments is shown. **(C, D)** Ba/F3-hgp130-hIL-6R and Ba/F3-hgp130 cells were incubated with the indicated concentrations of IL-6ts. STAT3 and ERK phosphorylation was determined by Western blotting as described in experimental procedures. One representative experiment out of three independent experiments is shown. **(E)** Ba/F3-hgp130-hIL-6R cells were incubated with the indicated concentrations of hIL-6 and hIL-11his and the IL-6R antibody tocilizumab (100 µg/ml) in the indicated concentrations. Cellular proliferation assay was determined as described in experimental procedures. One representative experiment out of two independent experiments is shown.

# Supplemental Figure 2

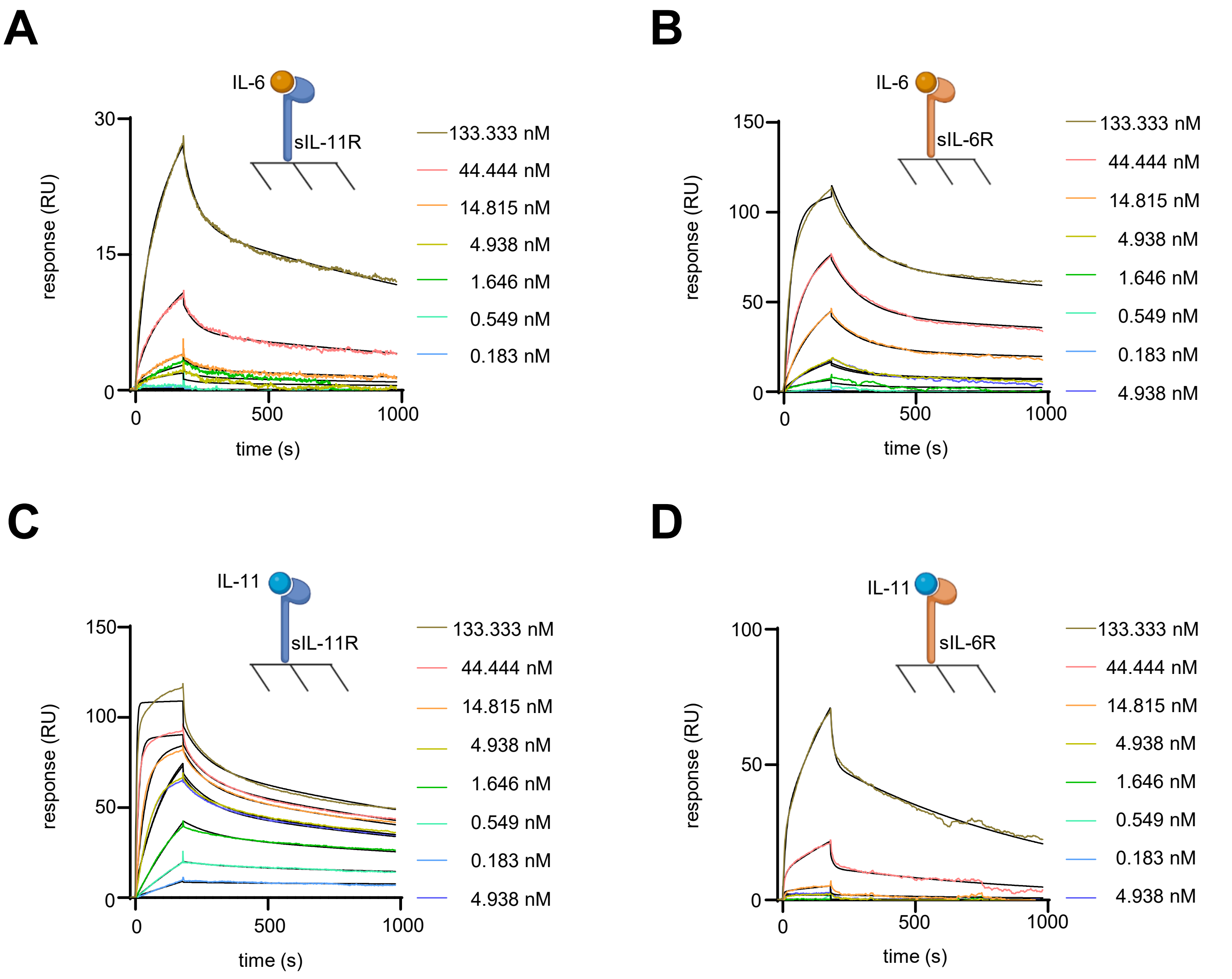


**Supplemental Figure S2. Binding kinetics of IL-6 and IL-11 to IL-6R and IL-11R, respectively. (A)** SPR analysis of hIL-6ts binding to shIL-11R. **(B)** SPR analysis of hIL-6ts binding to shIL-6R. **(C)** SPR analysis of hIL-11ts binding to shIL-11R. **(D)** SPR analysis of hIL-11ts binding to shIL-6R. **(A-D)** shIL-11R or shIL-6R were immobilized on a NTA chip and increasing concentrations of the cytokine were injected. Sensorgrams in response units (RU) over time are depicted as colored lines, and two-state reaction, local fit data are displayed as black lines.

# Supplemental Figure 3

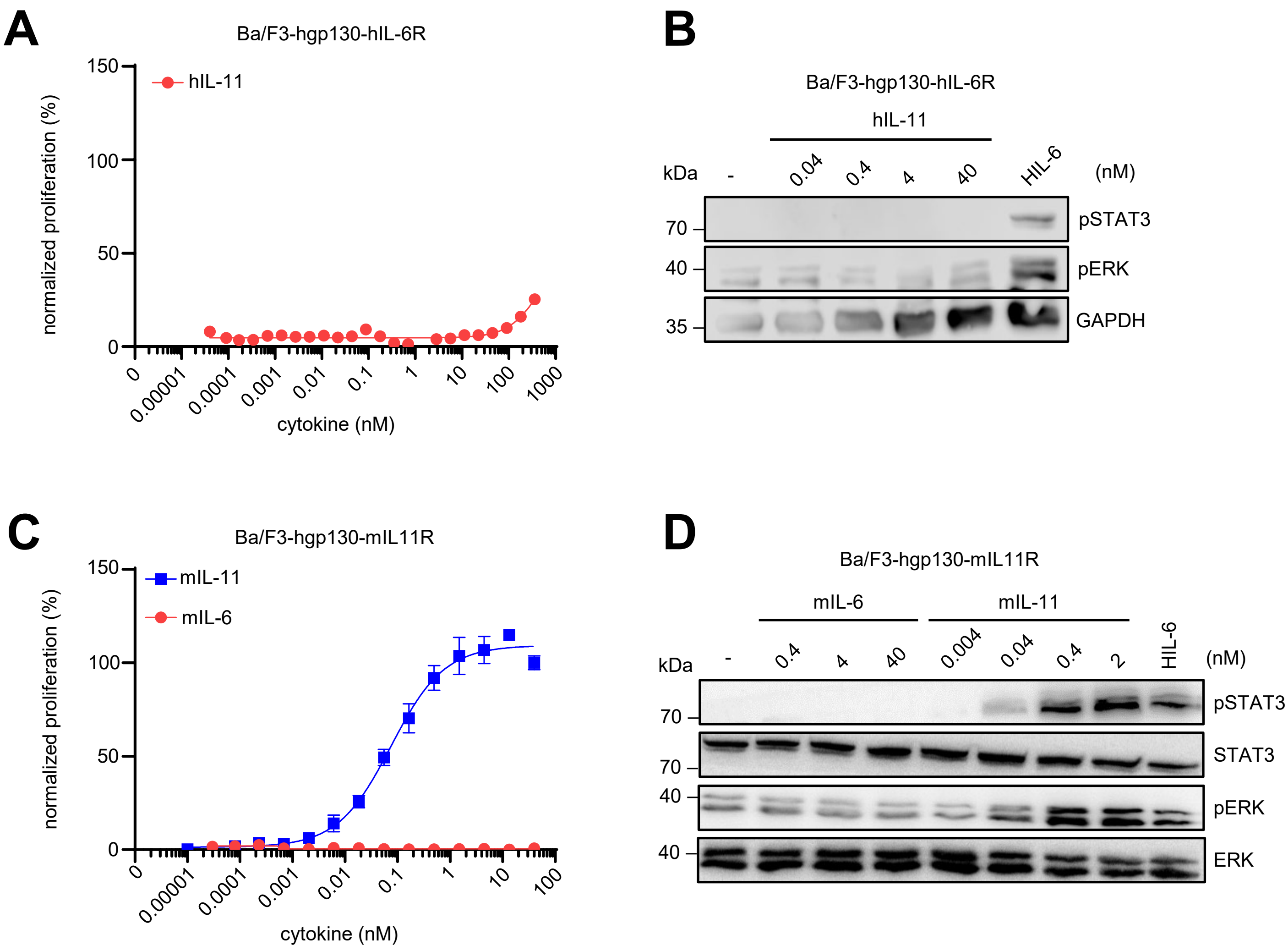


**Supplemental Figure S3: IL-11 did not induce proliferation of Ba/F3-hgp130-IL-6R cells. (A)** Ba/F3-hgp130-IL-6R cells were incubated with the indicated concentrations of IL-11ts. Cellular proliferation assay was performed in triplicate and determined after 72 h as described in experimental procedures. One representative experiment out of three independent experiments is shown. **(C)** Ba/F3-hgp130-mIL-11R cells were incubated with the indicated concentrations of mIL-11ts and mIL-6ts. Cellular proliferation assay was performed in triplicate and determined after 72 h as described in experimental procedures. One representative experiment out of three independent experiments is shown. **(D)** Ba/F3-hgp130-mIL-11R cells were incubated with the indicated concentrations of mIL-11ts and mIL-6ts or HIL-6 (10 ng/ml) for 20 min. STAT3 and ERK (phosphorylation) was determined by Western blotting as described in experimental procedures. One representative experiment out of three independent experiments is shown.

# Supplemental Figure 4

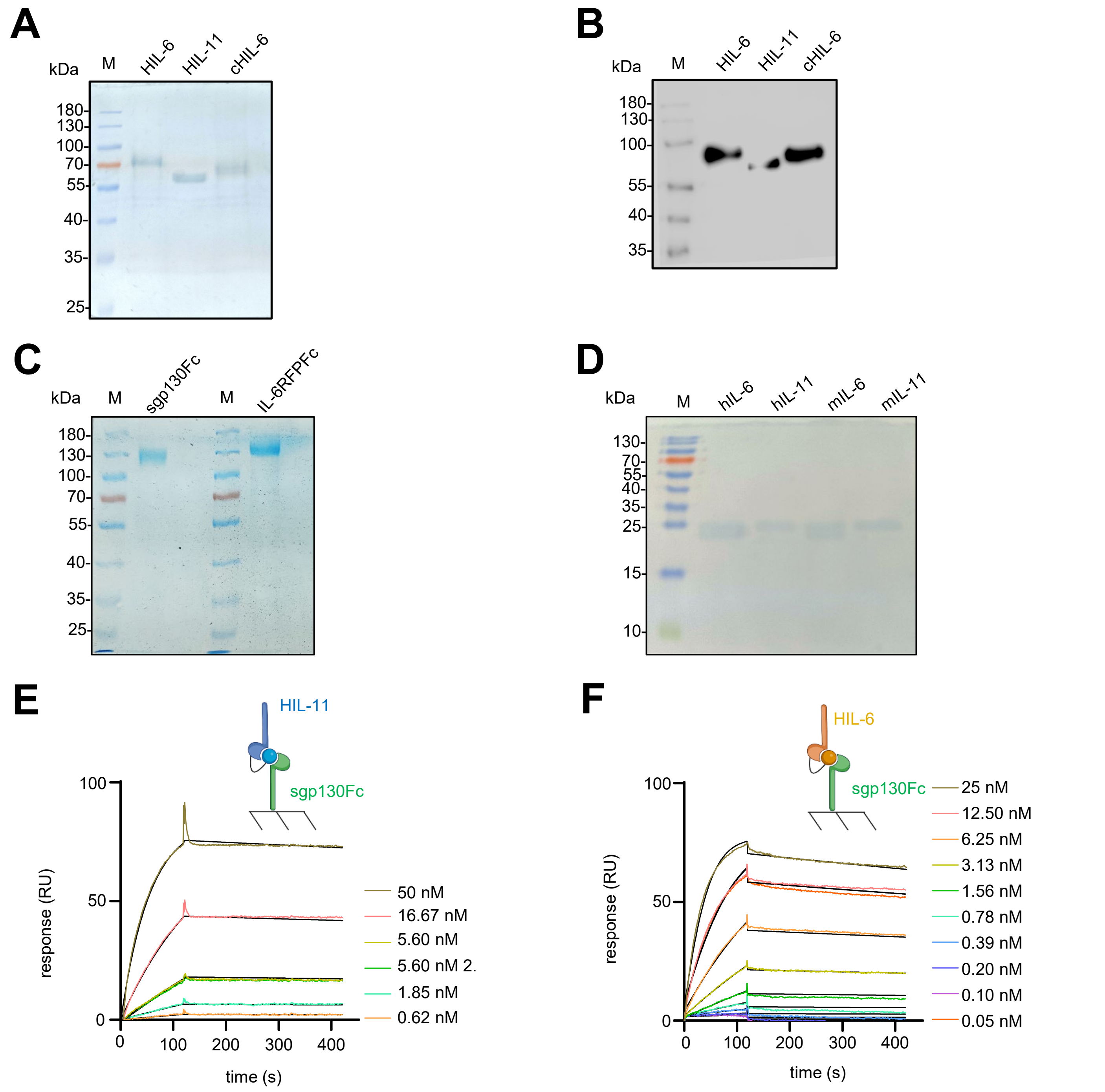


**Supplemental Figure S4. Validation of purified recombinant Proteins and binding kinetics of Hyper cytokines. (A)** Coomassie stained SDS-PAGE gel loaded with 2 µg of HIL-6ts, HIL-11ts and cHIL-6ts. **(B)** Western blotting of 100 ng of HIL-6ts, HIL-11ts, cHIL-6ts separated on an SDS-PAGE gel, blotted on NC Membrane and detected with the StrepMAB-Classic-HRP antibody. **(C)** Coomassie stained SDS-PAGE gel loaded with 3 µg of sgp130Fc and IL-6RFPFc. **(D)** Coomassie stained SDS-PAGE gel loaded with 3 µg of hIL-6ts. hIL-11ts, mIL-6ts and mIL-11ts. **(E)** SPR analysis of HIL-6 binding to sgp130Fc. Sgp130 was immobilized on a Protein A chip and increasing concentrations of the cytokine were injected. Sensorgrams in response units (RU) over time are depicted as colored lines, and global fit data are displayed as black lines. **(F)** SPR analysis of HIL-11 binding to sgp130Fc. Sgp130Fc was immobilized on a Protein A chip and increasing concentrations of the cytokine were injected. Sensorgrams in response units (RU) over time are depicted as colored lines, and global fit data are displayed as black lines.

# Supplemental Figure 5

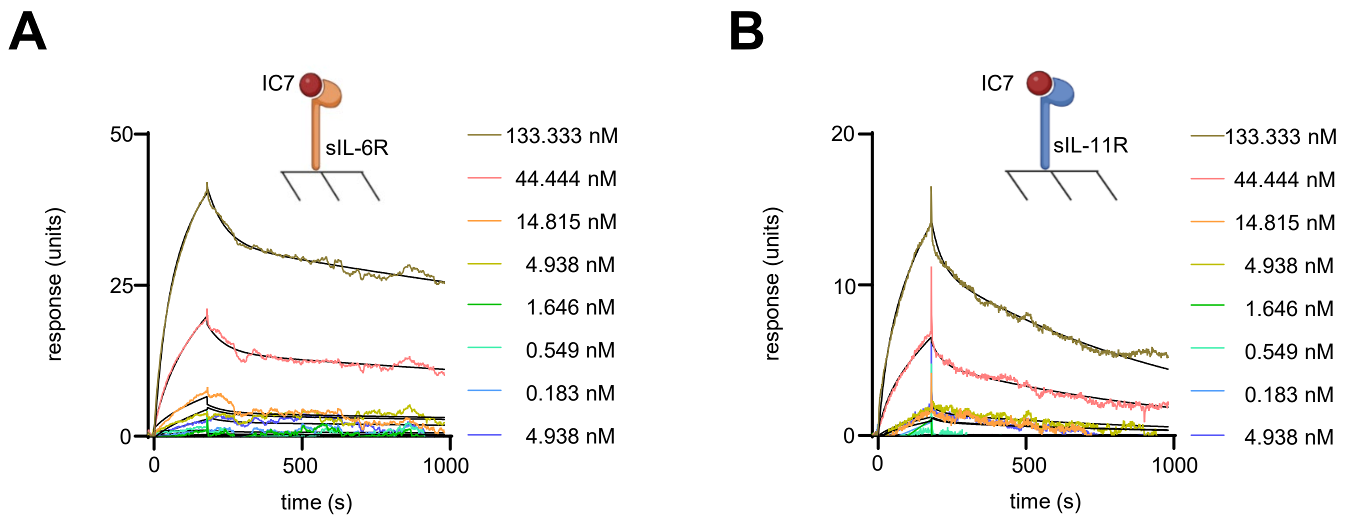


**Supplemental Figure S5. Binding kinetics of IC7 on IL-6R and IL-11R, respectively. (A)** SPR analysis of IC7ts binding to shIL-6R. **(B)** SPR analysis of IC7ts binding to shIL-11R. shIL-11R or shIL-6R were immobilized on a NTA chip and increasing concentrations of the soluble receptors were injected. Sensorgrams in response units (RU) over time are depicted as colored lines, and two-state reaction, local fit data are displayed as black lines.

# Supplemental Figure 6

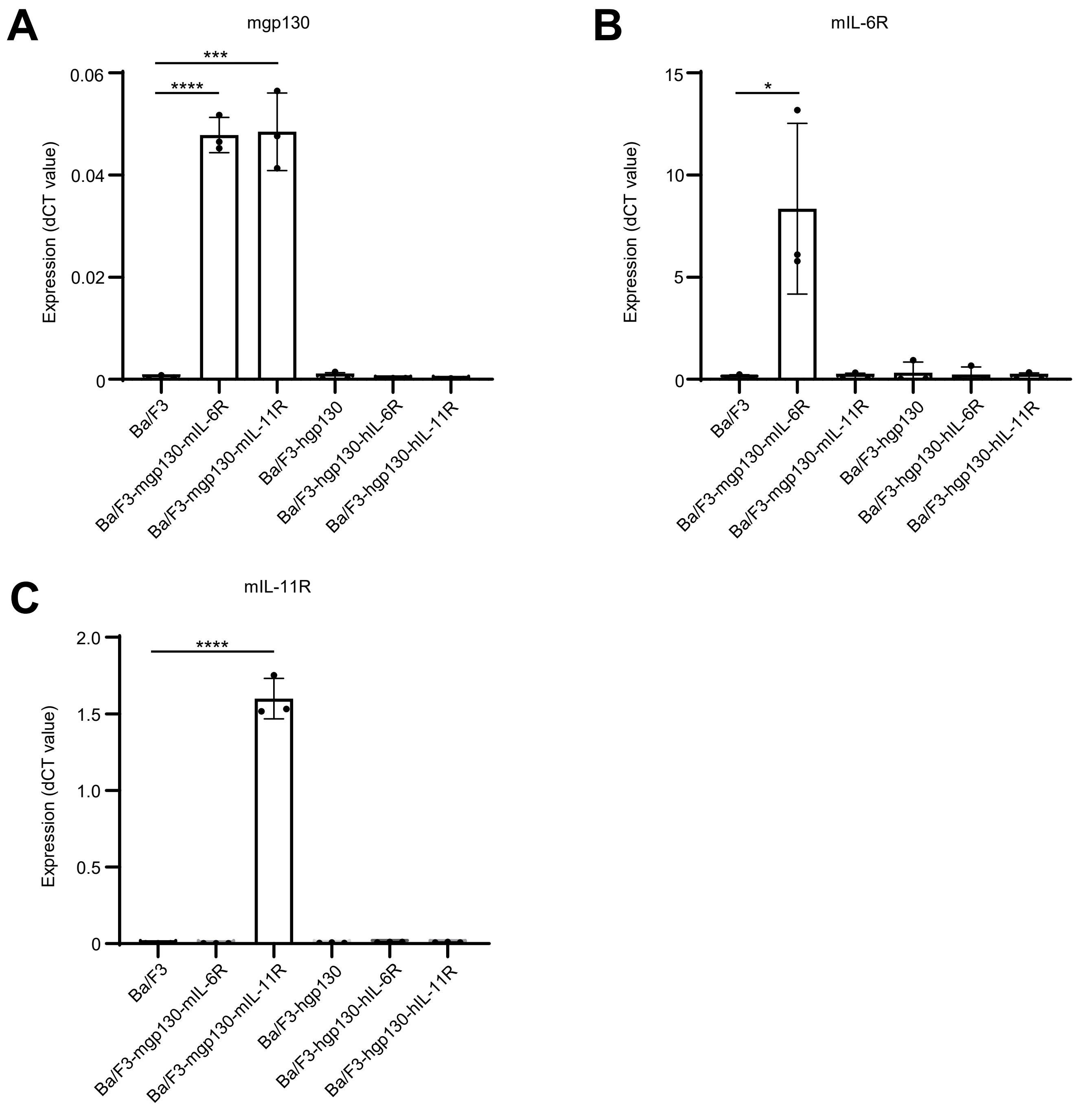


**Supplemental Figure S6. Detection of mRNAs for murine gp130, murine IL-6R and murine IL-11R in Ba/F3 cell lines by quantitative PCR. (A)** Detection of mgp130 mRNA in Ba/F3 cell lines. **(B)** Detection of mIL-6R mRNA in Ba/F3 cell lines. **(C)** Detection of mIL-11R mRNA in Ba/F3 cell lines. Statistical analysis used unpaired t test, $*p \leq 0.05$, $***p \leq 0.001$.
